# Loop Taping for Heel Fat Pad Syndrome: A Crossover Pilot Randomized Controlled Trial

**DOI:** 10.1002/jfa2.70229

**Published:** 2026-10-17

**Authors:** Alison H. Chang, Katrine Bruun Skov, Thomas Sørensen, Michael Skovdal Rathleff, Henrik Riel, Kristian Damgaard Lyng, Jens Lykkegaard Olesen, Marianne Christensen

**Affiliations:** ^1^ Department of Physical Therapy and Human Movement Sciences Northwestern University Feinberg School of Medicine Chicago Illinois USA; ^2^ Department of Physiotherapy and Occupational Therapy Aalborg University Hospital Aalborg Denmark; ^3^ Department of Physio‐ and Occupational Therapy Lillebaelt Hospital–University Hospital of Southern Denmark Vejle Denmark; ^4^ Department of Health Science and Technology Faculty of Medicine Aalborg University Aalborg Denmark; ^5^ Center for General Practice at Aalborg University Aalborg Denmark; ^6^ Department of Physiotherapy University College of Northern Denmark Aalborg Denmark; ^7^ Department of Orthopaedic Surgery Aalborg University Hospital Aalborg Denmark

**Keywords:** conservative management, foot pain, heel fat pad syndrome, plantar heel pain, randomized controlled trial, taping

## Abstract

**Objectives:**

Heel fat pad syndrome (HFPS) is a common but under‐investigated cause of plantar heel pain. No randomized controlled trials (RCTs) have evaluated targeted treatments. This study evaluated the immediate and short‐term effects of novel loop taping on pain, function, and fat pad thickness in adults with HFPS.

**Design:**

Two‐period crossover pilot RCT.

**Interventions:**

Participants completed two taping conditions (loop and control) in randomized order, separated by a 4‐to‐7‐day washout, with each participant serving as their own control.

**Outcome Measures:**

The primary outcome was immediate change in heel pain during the most painful weightbearing activity [0–10 numeric pain rating scale (NPRS)]. Secondary outcomes at 3 days post‐taping included changes in Foot Health Status Questionnaire (FHSQ) Pain and Function scores [0 (worst)–100 (best)] and in average 24‐h heel pain (NPRS). Mechanistic outcomes included heel fat pad thickness and pressure pain threshold (PPT). Linear mixed effects models estimated between‐condition differences.

**Results:**

Among 16 paired observations, loop taping produced a small, non‐significant between‐condition difference in change in immediate activity‐related pain (mean difference 0.8; 95% CI: −0.1 to 1.7; *p* = 0.10); 63% (loop) versus 25%–31% (control) met minimal clinically important difference (MCID) thresholds. Between‐condition differences favored loop taping for FHSQ Pain (20.7 points; 95% CI: 4.5 to 36.8; *p* = 0.02) and Function (11.3 points; 95% CI: −1.0 to 23.6; *p* = 0.09), exceeding MCIDs of 12.4 and 6.4 points. Fat pad thickness increased by 1.7 mm (95% CI: 1.2 to 2.3; *p* < 0.001) and correlated with improvement in FHSQ Pain. PPT did not differ between conditions.

**Conclusions:**

Loop taping showed favorable trends for reducing pain and improving function and significantly increased heel fat pad thickness. Although underpowered for definitive conclusions, several outcomes exceeded MCIDs. Findings support the potential of loop taping and inform primary outcome selection and design considerations for future, larger trials of this low‐cost, pathomechanics‐based intervention.

**Trail Registration:**

This RCT was registered at Clinicaltrialabas.gov (NCT05385796).

## 1 Introduction

Plantar heel pain is the most common musculoskeletal complaint of the foot [1], accounting for over 1 million outpatient visits annually in the US [2]. Its prevalence is estimated at 3.6%–7.3% in adults [3, 4] and 9.6%–11.1% in middle‐aged and older adults [5, 6]. Common causes include plantar fasciopathy, heel fat pad syndrome (HFPS), nerve irritation, and lumbar radiculopathy [7, 8, 9]. Prior investigations have predominantly focused on plantar fasciopathy, although HFPS is the second leading cause of plantar heel pain [5, 10, 11].

The heel fat pad consists of tightly packed adipose tissue encased in dense collagenous septa and elastin fibers [12], functioning to absorb shock and distribute plantar forces. A typical pad measures 12–28 mm in thickness [12, 13, 14]; but factors such as aging, injury, repetitive overloading, obesity, improper footwear, and comorbidities (e.g., diabetes) can cause atrophy, breakdown, or displacement [15, 16]. Loss of fat pad thickness and integrity reduces cushioning and load distribution, increasing focal pressure on periosteal and deep tissues [7, 8, 9] and leading to the characteristic deep central heel pain that worsens with weightbearing on hard surfaces and limits mobility.

Our recent systematic scoping review revealed a very limited evidence base for HFPS interventions [11], with no randomized controlled trials (RCTs) and a few quasi‐experimental studies adapted from protocols developed for plantar fasciopathy [17, 18]. Although the Clinical Practice Guideline (CPG) for plantar heel pain emphasizes the importance of differential diagnosis and recognizes HFPS as a distinct condition requiring tailored management [7], current recommendations remain largely extrapolated from plantar fasciopathy. A novel loop taping technique that encircles and centralizes the fat pad may provide a more targeted, pathomechanics‐based approach by increasing fat pad thickness and improving tissue resilience to compression. Given the absence of trial‐based evidence for HFPS‐specific interventions, this study aimed to generate preliminary data on the clinical and mechanistic effects of loop taping.

This two‐period crossover pilot RCT compared the immediate and short‐term effects of heel fat pad loop taping versus control taping on pain and function in individuals with HFPS. We hypothesized that loop taping would produce greater improvements in (1) the clinical outcomes of pain and function as well as (2) the mechanistic outcome of heel fat pad thickness than control taping.

## 2 Methods

### 2.1 Study Design

In this two‐period, crossover RCT, participants were randomized to one of two intervention sequences (AB or BA; A: heel fat pad loop taping; B: control taping) using block randomization (block size = 4). The random allocation sequence was generated in Microsoft Excel by a study investigator who was not involved in participant recruitment, eligibility assessment, or intervention delivery. Participant assignment to an intervention sequence occurred only after completion of the baseline assessment, confirmation of eligibility, and enrollment in the study. A 4‐to‐7‐day washout period between interventions minimized the carry‐over effect.

Ethical approval was obtained from the North Denmark Region ethics committee (N‐20210042). This RCT was registered at Clinicaltrials.gov (NCT05385796).

### 2.2 Study Participants

Participants were recruited from primary care, hospital outpatient clinics, private practices, and social media in the North Denmark Region. Eligibility was assessed by experienced musculoskeletal physiotherapists using a standardized screening protocol consisting of a preliminary screening interview, an in‐person clinical examination, and ultrasound assessment.

HFPS diagnosis was based on a standardized multimodal diagnostic approach integrating clinical history, symptom location, physical examination, and ultrasound findings to maximize diagnostic specificity. Participants were required to demonstrate the characteristic clinical presentation of HFPS, including central plantar heel pain, localized tenderness over the heel fat pad, and pain during weight‐bearing activities [11]. The physical examination included palpation of both the heel fat pad and plantar fascia to differentiate HFPS from plantar fasciopathy. Plantar fascia thickness was measured by musculoskeletal ultrasound at its calcaneal insertion using a standardized scanning protocol and interpreted in conjunction with the overall clinical evaluation. Participants with clinical findings suggestive of plantar fasciopathy were excluded regardless of plantar fascia thickness. A plantar fascia thickness ≥ 4 mm was considered an additional ultrasound finding associated with plantar fasciopathy and was interpreted in the context of the overall clinical evaluation [7, 19].

The inclusion criteria were: (1) ages 18–79 years, (2) plantar heel pain for ≥ 1 week and < 3 years, (3) tenderness on palpation of the heel fat pad, (4) worst central heel pain of ≥ 3 on a 0–10 numeric pain rating scale (NPRS) during the past week, and (5) pain ≥ 3 on NPRS during either 30‐sec single‐leg standing OR 20‐m barefoot walking. Exclusion criteria were: (1) primary diagnoses other than HFPS (e.g., plantar fasciopathy, posterior tibialis tendinopathy), (2) ultrasound‐measured plantar fascia thickness ≥ 4 mm, combined with clinical presentations of plantar fasciopathy, (3) BMI ≥ 35 kg/m^2^, (4) systemic inflammatory arthritis, (5) diabetes, (6) lumbar radiculopathy, (7) neurological conditions affecting gait and mobility, (8) recent taping for HFPS within the last month, and (9) prior heel surgery.

### 2.3 Intervention Protocol

This 2‐period crossover trial compared heel fat pad loop taping with control taping. Participants received both interventions in randomized order, separated by a 4‐to‐7‐day washout and were instructed to wear the tape for 3 days and to remove it after completing an online survey on day 3 post‐taping. Participants were told that both taping techniques had been used in clinical practice and that the study aimed to compare their effectiveness. To standardize delivery, 3 physiotherapists were trained in both taping techniques using a written protocol, demonstration video, and standardized instruction script. For bilateral heel pain, the more symptomatic heel was designated as the study index foot.

#### 2.3.1 Loop Taping

With the participant prone and feet over the table edge, the therapist cleaned the heel with alcohol wipes and applied skin preparation wipes to protect the skin and enhance tape adhesion. Using 1‐inch‐wide Elastikon elastic adhesive tape (Johnson & Johnson, USA), the therapist applied tension to encircle and bunch the heel fat pad, centralizing it to increase fullness and resilience to compression (Figure 1). The tape started at the distal midline of the fat pad, looped around the periphery of the pad, and crossed back over the starting point. Two to three overlapping loops were applied to ensure stability during weightbearing activities.

**FIGURE 1:**
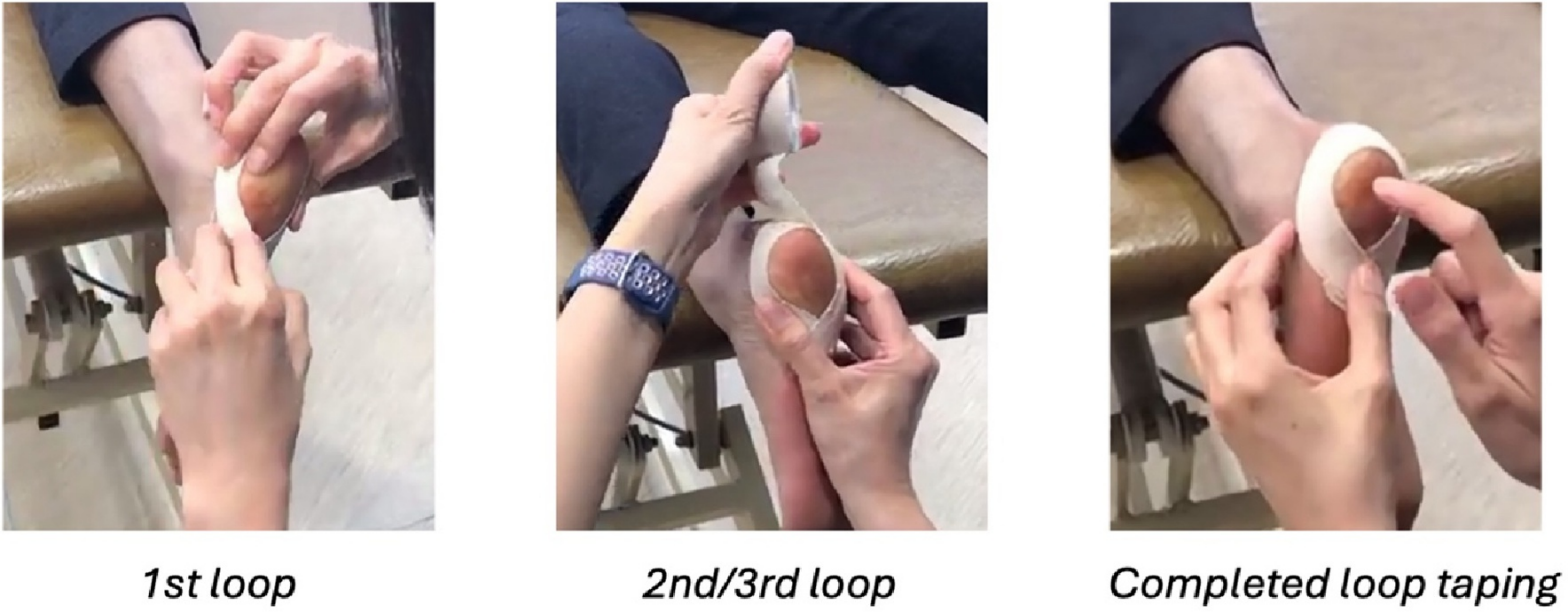
Application of heel fat pad loop taping. While prone with feet off the table, the participant's heel is cleaned and prepared with SKIN‐PREP. A physiotherapist applies 1‐inch‐wide Elastikon tape around the heel, bunching the heel fat pad to centralize and enhance its fullness and resistance to compression. Two to three overlapping loops are applied to support the heel during weightbearing. For control taping, the same procedure is followed, but the tape is applied loosely around the heel fat pad without pressure or attempt to bunch or centralize the pad. In both conditions, participants wore the taping for 3 days before removal.

#### 2.3.2 Control Taping

The control condition followed the same setup and materials as loop taping, except that the tape was applied loosely without tension or attempt to bunch or centralize the fat pad. Two to three overlapping loops were applied.

### 2.4 Clinical Outcomes

The primary outcome was heel pain intensity during the most painful weightbearing activity (30‐s single‐leg standing or 20‐m barefoot walking) measured at baseline and immediately after tape application using an 11‐point NPRS (0 = no pain, 10 = worst pain imaginable). The NPRS is recommended as a core outcome measure in clinical trials of chronic pain treatments [20] and demonstrates excellent reliability and validity in adults with chronic musculoskeletal pain [21, 22].

Secondary clinical outcomes, assessed at baseline and 3 days post‐taping, included Foot Health Status Questionnaire (FHSQ) Foot Pain and Foot Function scores and average heel pain by NPRS during the past 24 hours. The self‐administered FHSQ covers four domains (foot pain, foot function, footwear, general foot health) rated on 5‐point Likert scales [23]. Domain scores range from 0 (worst) to 100 (best), calculated using FHSQ software (https://www.fhsq.org/). A validated Danish version was used [24]. The FHSQ demonstrates strong reliability and validity in foot disorders [23, 25]. Consistent with prior studies of plantar heel pain [26, 27, 28, 29], only the Foot Pain and Foot Function scores were analyzed, which best reflect symptom severity and functional impact.

### 2.5 Mechanistic Outcomes

We assessed the immediate effect of taping on heel fat pad thickness and pain sensitivity. Thickness was measured using a 15–6 MHz ultrasound transducer (Sonosite X‐Porte, FUJIFILM SonoSite, USA) with participants prone and feet over the table edge. The fat pad was marked on the sagittal midline, and a transverse scan along this line measured the maximum distance between the calcaneus and skin. A generous layer of coupling gel was applied to optimize acoustic contact and to avoid tissue compression. The average of two measurements was analyzed [30]. Sonographic assessment of heel fat pad thickness demonstrates excellent reliability (ICC 0.81–0.96) [30].

Pain sensitivity was quantified using pressure pain threshold (PPT) at the most painful point on the heel fat pad, confirmed by palpation, with participants prone and their feet over the table edge. The examiner placed the 1‐cm^2^ probe of a handheld pressure algometer (FPX Algometer; Wagner, USA) perpendicular to the skin and gradually increased the pressure at a rate of 30 kPa/s. Participants were instructed to press a handheld switch at the first onset of pain. Three trials, separated by 30 seconds, were averaged for analysis [31]. The probe location was marked to ensure consistent placement between pre‐ and post‐intervention testing. PPT assessment has demonstrated good reliability in patients with plantar heel pain (ICC 0.80–0.94 intrarater; 0.75–0.92 interrater) [32].

### 2.6 Baseline Demographic, Health‐Related, and Clinical Characteristics

The collected variables included age, sex, body mass index (BMI), musculoskeletal pain in other body regions (yes/no), participation in leisure‐time sports activities (yes/no), current analgesic use for heel pain (yes/no), and previous treatment for heel pain (yes/no). Clinical characteristics included the mechanism of injury (insidious, acute, or unsure), symptom duration (months), and side of worse symptoms (right/left).

### 2.7 Sample Size Estimation

A priori calculation using G*Power 3.1 estimated that 18 participants were required to detect a 2‐point greater reduction in NPRS with loop taping versus control taping, assuming a within‐subject standard deviation of 2 points and 90% power with a 1‐sided alpha level of 0.05. To account for an anticipated 15% attrition rate, we planned to enroll 21 participants.

### 2.8 Statistical Analyses

Baseline demographic, health‐related, and clinical characteristics were summarized by means and standard deviations, medians and interquartile ranges, or counts and percentages, as appropriate. Carryover was assessed using a mixed‐effects model with sequence as the indicator and participant as a random effect.

We compared the effects of loop versus control taping using a linear mixed‐effects model. This model accounted for both within‐participant correlations of repeated observations and between‐participant variability. The model included the intervention group (loop vs. control taping) and period (first or second period) as fixed effects and participants as a random effect. Intervention effects were evaluated by comparing the changes from pre‐to post‐intervention in the clinical and mechanistic outcomes between the two taping groups. Effect sizes were expressed as standardized regression coefficients (*β*) derived from the linear mixed‐effects models. Statistical significance was set at a *p*‐value threshold of 0.05, and all analyses were conducted using R version 4.4.1.

### 2.9 Protocol Deviations

Recruitment challenges limited enrollment to 16 participants, below the planned sample size of 18. Given the smaller sample size, this study should be considered preliminary in nature. As the first pilot crossover RCT on HFPS, the findings provide important insights on feasibility, trial design, outcome selection, and sample size estimation for future definitive trials. Both clinical and mechanistic outcomes were analyzed with equal weight to explore treatment response patterns and potential signals of efficacy.

## 3 Results

### 3.1 Study Participants

Data were collected from 8th June 2022 to 10th December 2023. Of 75 individuals initially screened via phone or email, 49 were excluded, most commonly for suspected plantar fasciopathy, low pain severity at rest (NPRS < 3), and pain duration ≥ 3 years (Supporting Information S1: Table 1). Those meeting preliminary eligibility criteria underwent in‐person screening and physical examination, leading to 10 additional exclusions, mostly due to confirmed plantar fasciopathy. Sixteen participants enrolled and completed the study. Figure 2 details the study sample derivation process.

**FIGURE 2:**
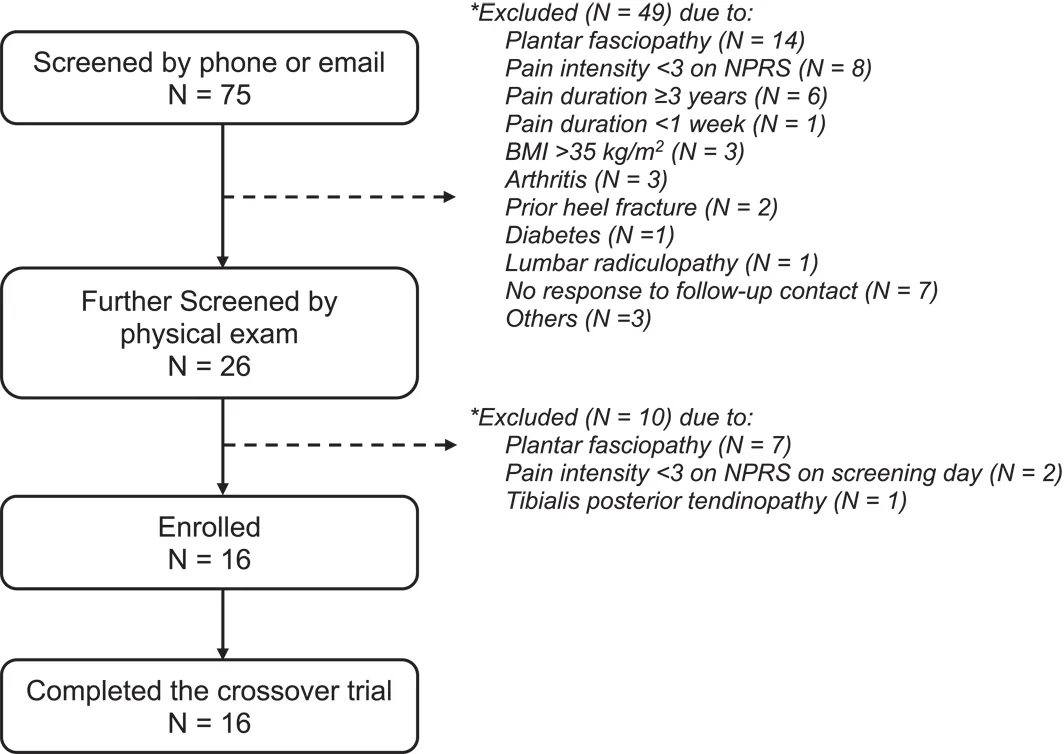
Participant screening and enrollment flow diagram. *Participants could have more than one exclusion reason. A single primary reason was assigned in this flow diagram according to the following hierarchy: (1) Alternative diagnosis: plantar fasciopathy or radiculopathy/neurological conditions; (2) Safety or contraindications: BMI ≥ 35 kg/m², diabetes, inflammatory arthritis, or prior fracture/injury/surgery; (3) Failed pain criteria: symptom duration ≥ 3 years or pain rating < 3 at rest or during activity; (4) Procedural: taping within the past month; (5) Administrative: no response to follow‐up contact; (6) Other: miscellaneous reasons not captured above.

Table 1 summarizes participant characteristics. The sample included 16 participants with HFPS, with a mean age of 44.8 years (SD: 10.5 years) and a mean BMI of 27.6 kg/m^2^ (SD: 3.6 kg/m^2^); 63% were women. Mean heel fat pad thickness was 17.6 mm (SD: 3.0 mm). The average symptom duration was 15.9 months with 88% reporting symptoms ≥ 3 months. Participants reported moderate‐to‐high levels of heel pain and moderate impairments in foot pain and function. No carry‐over effects were observed for any outcome measure, indicating that the 4‐to‐7‐day washout period was adequate.

**TABLE 1:** Baseline characteristics of study participants (*n* = 16).

Baseline characteristics	Mean (SD)	Median (IQR)	*N* (%)
Age (year)	44.8 (10.5)	48.0 (14.3)	
Sex (female)			10 (63%)
BMI (kg/m^2^)	27.6 (3.6)	27.8 (5.6)	
MSK pain in other body regions (yes)			11 (69%)
Participate in leisure‐time sports activities			8 (50%)
Current analgesic use for heel pain (yes)			4 (25%)
Previous treatment of heel pain (yes)			9 (56%)
Mechanism of injury			
Insidious			10 (63%)
Acute onset			5 (31%)
Unsure			1 (6%)
Symptom duration (month)	15.9 (13.9)	9.0 (19.6)	
Study foot (right side)			7 (44%)
Plantar fascia thickness (mm)	3.48 (0.45)	3.60 (0.60)	
Heel fat pad thickness (mm)	17.6 (3.0)	18.3 (4.3)	
Heel pain during 20‐m walk (range: 0–10)	4.8 (0.8)	5.0 (1.0)	
Heel pain during 30‐sec stand (range: 0–10)	4.1 (1.4)	3.5 (2.0)	
Worst heel pain in past 24 hours (range: 0–10)	6.6 (2.0)	6.5 (3.0)	
Foot Health Status Questionnaire (FHSQ) *(range: 0‐100, higher is better)*			
Pain	41.1 (17.3)	40.6 (26.9)	
Function	49.2 (20.1)	50.0 (34.4)	
Footwear	37.5 (21.3)	33.3 (27.1)	
General foot health	44.8 (31.9)	42.5 (50.6)	

### 3.2 Immediate Post‐Taping Change in Heel Pain During the Most Painful Weightbearing Activity (Primary Outcome)

Loop taping produced a small, non‐significant between‐condition difference in the primary outcome (mean difference = 0.8 points; 95% CI: −0.1, 1.7; *p* = 0.10; standardized treatment effect (*β*) = 0.30; Figure 3A). Average pain reduction was 0.8 (SD: 1.4) with loop taping versus no change (SD: 1.2) with control. Using MCID thresholds for improvement (≥ 15% or ≥ 1‐point reduction) [26, 33, 34], 63% of participants met both criteria with loop taping, compared to 25% (≥ 15% reduction) and 31% (≥ 1‐point reduction) with control.

**FIGURE 3:**
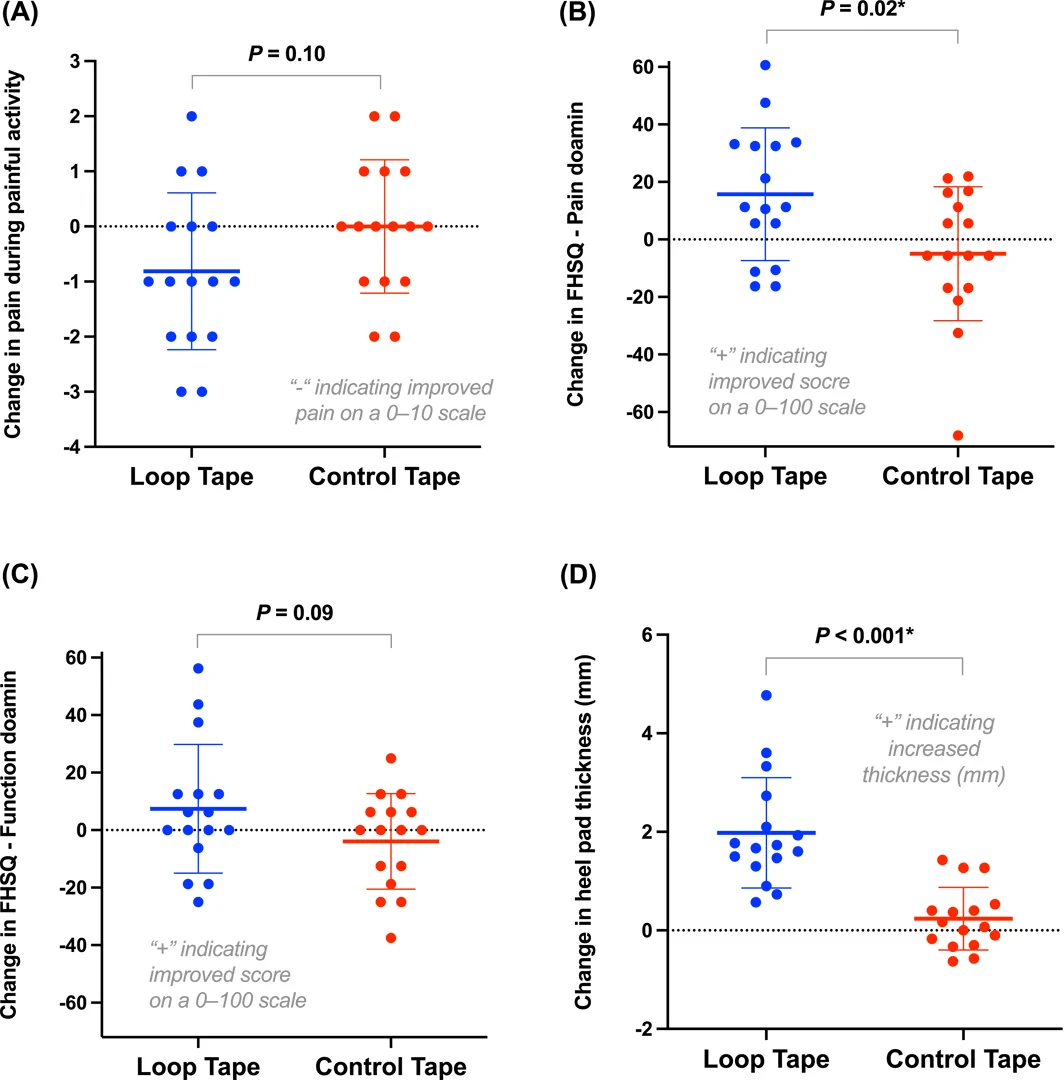
Effects of loop taping on 4 outcome measures. Each dot represents an individual participant; bars indicate group means and standard deviations (blue = loop taping, red = control taping). (A) Immediate change (from baseline to immediately post‐taping) in heel pain intensity during each participant's most painful weightbearing activity: mean difference of 0.8 points on a 0‐10 scale (95% CI: −0.1, 1.7; *P* = 0.10). (B) Short‐term change (from baseline to 3‐day post‐taping) in FHSQ Pain scores: mean difference of 20.7 points on a 0‐100 scale (95% CI: 4.5, 36.8; *P* = 0.02). (C) Short‐term change (from baseline to 3‐day post‐taping) in FHSQ Function scores: mean difference of 11.3 points on a 0–100 scale (95% CI: −1.0, 23.6; *P* = 0.09). (D) Immediate change (from baseline to immediately post‐taping) in heel fat pad thickness (mm): mean difference of 1.7 mm (95% CI: 1.2, 2.3; *p* < 0.001).

### 3.3 Three‐Day Post‐Taping Change in FHSQ Pain and Function

Loop taping significantly improved FHSQ Pain compared to control (mean difference = 20.7 points; 95% CI: 4.5, 36.8; *p* = 0.02; standardized treatment effect (*β*) = 0.42; Figure 3B). The average FHSQ Pain improved by 15.7 points with loop taping versus a 5.0‐point decline with control. This difference exceeds the MCID of 12.4 points [26], indicating a clinically meaningful improvement. Loop taping modestly improved FHSQ Function (mean difference = 11.3 points; 95% CI: −1.0, 23.6; *p* = 0.09; standardized treatment effect (*β*) = 0.28; Figure 3C). Function improved by 7.4 points with loop taping versus a decline of 3.9 points with control. This difference exceeds the MCID of 6.4 points [26].

### 3.4 Three‐Day Post‐Taping Change in Average Pain During the Past 24 Hours

Loop taping slightly reduced pain (mean difference = 1.1 points; 95% CI: −0.8, 2.9; *p* = 0.28; standardized treatment effect (*β*) = 0.19). Average pain reduction was 1.2 with loop taping versus 0.2 with control. Responder rates were 44% (≥ 15% reduction) or 56% (≥ 1‐point reduction) with loop taping, compared to 31% (for both thresholds) with control.

### 3.5 Immediate Post‐Taping Change in Heel Fat Pad Thickness and PPT (Mechanistic Outcomes)

Loop taping significantly increased fat pad thickness versus control (mean difference = 1.7 mm; 95% CI: 1.2, 2.3; *p* < 0.001; standardized treatment effect (*β*) = 0.70; Figure 3D). The average thickness increased by 2.0 mm (SD: 1.1 mm) with loop taping versus 0.2 mm (SD: 0.6 mm) with control. Thickness change showed a moderate positive correlation with improvement in FHSQ Pain (Pearson's *r* = 0.56, *p* = 0.02; Spearman's *ρ* = 0.56, *p* = 0.03) (Figure 4), but not with activity‐related pain (Pearson's *r* = −0.24, *p* = 0.38; Spearman's *ρ* = 0.01, *p* = 0.99). Change in PPT did not differ between conditions (mean difference = 22.2 kPa; 95% CI: −40.1, 84.6; *p* = 0.49; standardized treatment effect (*β*) = 0.13).

**FIGURE 4:**
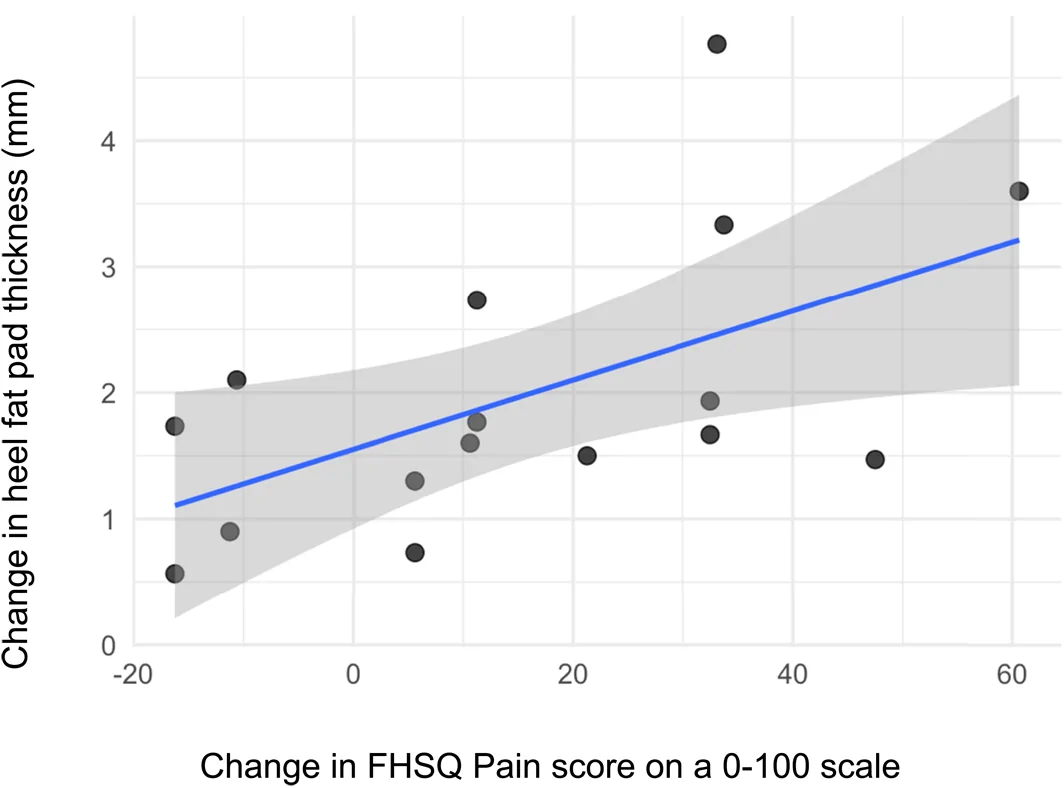
Association between changes in FHSQ Pain and changes in heel fat pad thickness. Each point represents an individual participant; blue line shows least‐squares linear fit with 95% CI. There was a moderate positive correlation (Pearson *r* = 0.56, *p* = 0.02; Spearman *ρ* = 0.56, *p* = 0.03), indicating that greater increases in heel fat pad thickness corresponded to larger improvements in FHSQ Pain.

Supporting Information S1: Table 2 presents the pre‐ and post‐intervention mean (SD) values for all clinical and mechanistic outcomes following loop taping and control taping.

## 4 Discussion

This crossover pilot RCT is the first to evaluate an HFPS‐specific intervention. Loop taping significantly increased heel fat pad thickness, supporting its proposed biomechanical action of centralizing and stabilizing the fat pad. Loop taping also significantly reduced FHSQ Pain, exceeding the established MCID for clinically meaningful improvement. Although between‐group differences in activity‐related pain and FHSQ Function did not reach statistical significance, responder proportions favored loop taping, and the difference in FHSQ Function change also exceeded the MCID. Given the small sample size, the trial was underpowered for definitive conclusions, but the observed short‐term benefits and responder rates support conducting larger, adequately powered trials with longer follow‐up. These findings provide preliminary support for this pathomechanics‐based intervention for HFPS, informing feasibility and outcome selection for future definitive trials.

Loop taping produced a small, non‐significant between‐condition difference in change in activity‐related pain. Although not statistically significant (*p* = 0.10), the consistent direction of effect and the higher responder rates (63% vs. 25% achieving ≥ 15% pain reduction) suggest a possible clinically relevant benefit. The wide confidence interval and non‐significant *p*‐value likely reflect limited statistical power rather than a true absence of effect. Indeed, a post hoc power calculation indicated that with a sample size of 16, the study had only 47% power to detect the observed effect, underscoring the need for a larger, adequately powered trial.

Loop taping improved both FHSQ Pain and function beyond their respective MCIDs, with a statistically significant benefit for pain (*p* = 0.02) but not function (*p* = 0.09). These results support the FHSQ as a sensitive and clinically meaningful measure for HFPS. Similar patterns have been observed in plantar fasciopathy trials [27, 29, 35, 36] where pain scores appeared to respond earlier and more consistently than functional measures, which may require longer follow‐up periods or larger sample sizes to detect significant changes.

Compared with single‐item pain ratings, the FHSQ Pain domain offers a broader representation of patients' symptom experiences across weightbearing tasks and daily activities [7, 23]. This may explain why FHSQ Pain demonstrated a clear, clinically meaningful improvement, whereas the single‐activity NPRS changed only modestly. In line with the proposed biomechanical effect, greater increases in fat pad thickness were correlated with larger improvements in FHSQ Pain, suggesting a link between mechanical restoration and pain relief. Together, these findings underscore the FHSQ Pain domain as a sensitive, mechanistically relevant outcome for symptom improvement in HFPS and support its use as a primary endpoint in future trials.

Loop taping significantly increased heel fat pad thickness compared with control (between‐condition difference = 1.7 mm; mean increase: 2.0 vs. 0.2 mm), well beyond the reported standard error of measurement (0.15–0.52 mm) [37]. This supports its intended biomechanical action of centralizing and stabilizing the fat pad, potentially improving load dissipation during weightbearing. The association between increased thickness and reduced FHSQ Pain further suggests a mechanical contribution to symptom relief. In contrast, PPT did not change, suggesting effects on tissue mechanics rather than pain sensitivity and supporting a predominantly biomechanical mechanism of symptom change.

This study has notable strengths. This is the first RCT to investigate an HFPS‐specific intervention, addressing a major gap in the literature. The use of a robust crossover design minimized between‐participant variability by having each participant serve as their own control. Both mechanistic (fat pad thickness and pain sensitivity) and clinical (pain and function) outcomes were assessed, providing a more complete understanding of the intervention's effects. This pilot study provides valuable preliminary evidence supporting the potential role of loop taping in managing this under‐researched condition.

There are several key limitations. First, the small sample size limited statistical power to detect effects in some clinical outcomes. Second, recruitment proved challenging due to diagnostic overlap with plantar fasciopathy and other sources of heel pain, which may have contributed to slower enrollment and limited generalizability. Furthermore, because no universally accepted diagnostic criteria currently exist for HFPS, some diagnostic uncertainty remains despite our use of a standardized multimodal diagnostic approach and conservative eligibility criteria. Although these procedures were intended to minimize inclusion of participants with concomitant plantar fasciopathy, residual diagnostic misclassification cannot be completely excluded. Although MRI may have provided additional characterization during participant screening by identifying plantar fascia pathology, heel fat pad abnormalities, and other causes of plantar heel pain, it was beyond the scope of this pilot study. Additionally, MRI is not considered the gold standard for diagnosing HFPS, and no universally accepted imaging reference standard currently exists. Third, the short follow‐up period captured only immediate and short‐term responses, precluding conclusions about sustained effects or long‐term adherence. Finally, as a pilot study, the trial was primarily intended to assess feasibility and explore preliminary effects rather than to provide definitive evidence of effectiveness. These limitations underscore the need for cautious interpretation but also offer useful information on diagnostic approaches, outcome selection, recruitment feasibility, and sample size estimation for future larger‐scale trials.

Loop taping is a low‐cost, non‐invasive intervention that can be readily incorporated into routine care and may offer short‐term mechanical and symptomatic benefits for HFPS. These early benefits may help patients better tolerate weightbearing activities while longer‐term strategies, such as footwear and activity modifications, are implemented, although confirmation in larger and longer‐term trials is still needed.

Future studies should examine the durability of loop taping beyond the short‐term window in this pilot, including effects with repeated or prolonged use. Additional research is also needed on the feasibility of self‐application outside the clinical environment. Comparative effectiveness trials should evaluate loop taping alone or in combination with other conservative strategies (e.g., heel cups, activity modification) to determine its relative and additive value. Broader recruitment, including older adults, individuals with higher BMI or comorbidities, and those with concomitant plantar fasciopathy, will enhance generalizability and real‐world feasibility.

## Author Contributions

**Alison H. Chang:** conceptualization, methodology, formal analysis, investigation, writing – original draft, writing – review and editing, visualization, project administration. **Katrine Bruun Skov:** methodology, investigation, writing – review and editing. **Thomas Sørensen:** methodology, investigation, writing – Review and editing. **Michael Skovdal Rathleff:** conceptualization, methodology, writing – review and editing. **Henrik Riel:** methodology, investigation, writing – review and editing. **Kristian Damgaard Lyng:** methodology, investigation, writing – review and editing. **Jens Lykkegaard Olesen:** methodology, investigation, writing – review and editing. **Marianne Christensen:** conceptualization, methodology, writing – review and editing, project administration, supervision.

## Funding

The authors have nothing to report.

## Conflicts of Interest

The authors declare no conflicts of interest.

## Supporting information


Supporting Information S1

Supporting Information S2

## Data Availability

The data that support the findings of this study are available on request from the corresponding author.
