# Supplementary material for: Loop Taping for Heel Fat Pad Syndrome: A Crossover Pilot Randomized Controlled Trial
Source: J Foot Ankle Res. 2026 Oct 17;19(4):e70229. doi: 10.1002/jfa2.70229 (PMC13676420; doi:10.1002/jfa2.70229)
Supplement: Supplementary file 1 [file JFA2-19-e70229-s001.docx]

**Supplemental Table 1: Reasons for exclusion among 49 screened candidates by phone or email**

| **ID of screened candidates** | **Pain duration ≥3 years** | **Pain intensity <3 at rest** | **Pain intensity <3 during weightbearing activities** | **Presence of plantar fasciopathy** | **Lumbar radiculopathy** | **BMI >35 kg/m^2^** | **Diabetes (DM) or inflammatory arthritis** | **Prior heel fracture** | **Other** | **No reply to**  **follow-up contact** | **Tape**  **within past month** |
| --- | --- | --- | --- | --- | --- | --- | --- | --- | --- | --- | --- |
| 1 |  | **x** | **x** |  |  |  |  |  |  |  |  |
| 2 |  |  |  | **x** | **x** |  |  |  |  |  |  |
| 4 |  | **x** | **x** |  |  |  |  |  |  |  |  |
| 5 |  | **x** |  |  |  |  |  |  |  |  |  |
| 8 |  |  |  | **x** |  |  |  |  |  |  |  |
| 10 | **x** |  |  |  |  |  |  |  |  |  |  |
| 11 |  |  |  |  |  | **x** | **x**  DM |  |  |  |  |
| 14 |  |  |  | **x** |  |  |  |  |  |  |  |
| 16 |  |  |  |  |  |  | **x**  DM |  |  |  |  |
| 17 |  |  |  |  |  |  |  |  |  | **x** |  |
| 18 | **x** |  |  |  |  |  |  |  |  |  |  |
| 19 |  |  |  |  |  | **x** |  |  |  |  |  |
| 20 |  |  |  |  |  |  |  |  | **x** |  |  |
| 21 |  |  |  |  |  |  |  | **x** |  |  |  |
| 22 |  |  |  |  |  |  |  | **x** |  |  |  |
| 23 |  | **x** |  |  |  |  |  |  |  |  | **x** |
| 25 | **x** |  |  |  |  |  |  |  |  |  |  |
| 26 |  |  |  | **x** |  |  |  |  |  |  |  |
| 27 |  |  |  |  | **x** |  |  | **x** |  |  |  |
| 29 |  |  |  | **x** | **x** |  |  |  |  |  |  |
| 30 |  |  |  |  |  |  |  |  |  | **x** |  |
| 31 |  |  |  | **x** |  |  |  |  |  |  |  |
| 33 |  |  |  | **x** |  |  |  |  |  |  |  |
| 35 |  |  |  | **x** |  | **x** |  |  |  |  |  |
| 37 |  | **x** |  | **x** |  |  |  |  |  |  |  |
| 38 |  | **x** |  |  |  |  |  |  |  |  |  |
| 39 |  |  |  | **x** |  |  |  |  |  |  |  |
| 40 |  |  |  | **x** |  |  |  |  |  |  |  |
| 41 |  |  |  |  |  |  |  |  | **x**  Haglunds? |  |  |
| 42 | **x**  (pain duration <1 week |  |  |  |  |  |  |  |  |  |  |
| 45 |  |  |  | **x** |  |  |  |  |  |  |  |
| 46 |  |  |  |  |  |  |  |  |  | **x** |  |
| 47 |  |  |  |  |  |  | **x**  arthritis |  |  |  |  |
| 49 | **x** |  |  |  |  |  |  |  |  |  |  |
| 50 |  |  |  |  |  |  |  |  |  | **x** |  |
| 52 |  | **x** |  |  |  |  |  |  |  |  |  |
| 53 |  |  |  |  |  |  | **x**  ankylosing  spondylitis |  |  |  |  |
| 54 |  |  |  |  |  |  | **x**  arthritis |  |  |  |  |
| 56 |  | **x** |  |  |  |  |  |  |  |  |  |
| 57 |  |  |  |  |  |  |  |  | **x**  pain around midfoot and arch |  |  |
| 59 |  |  | **x** |  |  | **x** |  |  |  |  |  |
| 60 |  | **x** |  |  |  |  |  |  |  |  |  |
| 64 |  | **x** | **x** | **x** |  |  |  |  |  |  |  |
| 66 |  |  |  | **x** |  |  |  |  |  |  |  |
| 67 | **x** |  |  |  |  |  |  |  |  |  |  |
| 68 |  |  |  |  |  |  |  |  |  | **x** |  |
| 71 | **x** |  |  |  |  |  |  |  |  |  |  |
| 74 |  |  |  |  |  |  |  |  |  | **x** |  |
| 75 |  |  |  |  |  |  |  |  |  | **x** |  |
| **Total** | **7** | **10** | **4** | **14** | **3** | **4** | **5** | **3** | **3** | **7** | **1** |

*Abbreviation: NPRS = Numeric Pain Rating Scale (range: 0-10); DM = Diabetes Mellitus*

**Supplementary Table 2. Pre- and post-intervention values for clinical and mechanistic outcomes**

*Values are presented as mean (SD).*

|  | **Loop Taping** | | **Control Taping** | |
| --- | --- | --- | --- | --- |
| **Outcome** | Pre-intervention | Post-intervention | Pre-intervention | Post-intervention |
| *3-day post-taping* | | | | |
| FHSQ Pain  (0-100, higher scores indicating better foot health) | 42.9 (19.0) | 58.6 (22.6) | 50.0 (19.9) | 45.0 (19.5) |
| FHSQ Function  (0-100, higher scores indicating better foot health) | 53.9 (21.6) | 61.3 (23.2) | 55.5 (21.6) | 51.6 (19.4) |
| Average heel pain during the previous 24 hours  (0–10, higher scores indicating worse pain) | 6.2 (2.0) | 5.1 (2.8) | 5.8 (2.2) | 5.5 (2.8) |
| *Immediate post-taping* | | | | |
| Heel pain intensity during most painful weightbearing activity  (0–10, higher scores indicating worse pain) | 4.7 (0.9) | 3.9 (1.1) | 4.4 (1.8) | 4.4 (1.9) |
| Heel fat pad thickness (mm) | 17.7 (2.8) | 19.7 (3.4) | 17.7 (2.5) | 17.9 (2.3) |
| Pressure pain threshold (kPa) | 385.0 (126.0) | 426.2 (145.9) | 357.1 (126.4) | 376.1 (170.1) |

*Abbreviations****:*** *NPRS = Numeric Pain Rating Scale; FHSQ = Foot Health Status Questionnaire; SD = standard deviation*
